# Appraising convergent validity of patient-reported outcome measures in systematic reviews: constructing hypotheses and interpreting outcomes

**DOI:** 10.1186/s13104-016-2034-2

**Published:** 2016-04-19

**Authors:** Inger L. Abma, Maroeska Rovers, Philip J. van der Wees

**Affiliations:** Radboud Institute of Health Sciences, Radboud University Medical Center, IQ healthcare, PO box 9101, huispost 114, 6500 HB Nijmegen, The Netherlands; Departments for Health Evidence and Operating Rooms, Radboud Institute of Health Sciences, Radboud University Medical Center, Nijmegen, The Netherlands

## Abstract

**Purpose:**

Convergent validity is one type of validity that is commonly assessed for patient-reported outcome measures (PROMs). It is assessed by means of “hypothesis testing”: determining whether the scores of the instrument under study correlate with other instruments in the way that one would expect. Authors of systematic reviews on measurement properties for PROMs may encounter validation articles which do not state hypotheses by which convergent validity can be tested. The information in these articles can therefore not be readily used to determine the adequacy of convergent validity. We suggest that in these cases, reviewers construct their own hypotheses. However, constructing hypotheses and interpreting outcomes is not always straightforward, and we wish to aid reviewers based on our own recent experiences with a systematic review on measurement properties.

**Recommendations:**

We have the following recommendations for authors of a systematic review on measurement properties who wish to construct hypotheses for convergent validity: take an active role in judging the suitability of the comparator instruments of validation articles; be transparent about which hypotheses were constructed, the underlying assumptions on which they are based, and whether they were constructed by the authors of the validation article or by the reviewer; discuss unmet hypotheses, especially if convergent validity is judged to be inadequate; and when synthesizing data, add up the results of all hypotheses for one instrument, rather than judging convergent validity per study.

## Background

Questionnaires about patients’ health and functioning filled out by the patient, also known as patient-reported outcome measures (PROMs), should be validated to ensure that they measure the topic (“construct”) that they aim to measure (validity), and that they do this in a reliable way (reliability). There are several different aspects of validity and reliability that can be assessed to determine the quality of a PROM. The international Delphi panel of COnsensus-based Standards for the selection of health Measurement Instruments (COSMIN) reached consensus on a comprehensive terminology of these measurement properties, as well as on the content of the first user-friendly quality checklist for validation studies [1–3]. The COSMIN checklist and guidelines are frequently utilized: a search in PubMed for COSMIN shows 50 systematic reviews on measurement properties using the COSMIN checklist in 2015 alone.

One aspect of validity is construct validity, which is the degree to which the scores of a PROM are consistent with hypotheses, based on the assumption that the PROM validly measures the construct to be measured [4–7]. Convergent validity, a subtype of construct validity, verifies whether the scores of the instrument under study “make sense” in relation to the scores of other, related instruments. Scores should correlate with scores of other instruments to the degree that one would expect. Assessing convergent validity is an iterative process: the more hypotheses are tested, the stronger the evidence towards the instrument being valid. Convergent validity is generally considered adequate if >75 % of hypotheses are correct, or if a correlation with an instrument measuring the same construct is >0.50. The exact values of these cut-off points may be arbitrary, but they provide guidance when judging whether convergent validity is adequate. Furthermore, correlations with related constructs should be higher than with unrelated constructs [4, 8].

When performing a systematic review on measurement properties, assessing and summarizing the data for convergent validity is often less straightforward than for many other measurement properties. Authors of validation studies do not always construct hypotheses when studying convergent validity: many studies present only correlation sizes, without interpreting these or using them to test expectations. Based on the COSMIN guidelines, this data cannot be readily used in a systematic review. Therefore, the authors of a recent systematic review [9] decided to construct their own hypotheses for convergent validity. In our own recent systematic review on the measurement properties of PROMs for obstructive sleep apnea (OSA) [10], we followed their example.

We believe that constructing hypotheses for convergent validity should become more common in systematic reviews for measurement properties in which the included studies do not present their own hypotheses. However, there are certain issues that will arise when approaching hypothesis testing this way, which include: how to deal with unsuitable or low-quality comparator instruments; the different ways in which hypotheses can be constructed; interpreting the results and synthesizing the evidence, which are a general issues regardless of the approach. These issues have not yet been discussed in the literature. The aim of this paper is to provide an overview of these issues regarding convergent validity, and to start a discussion on how they can best be handled in future systematic reviews. Additionally, we believe that the considerations of this paper will aid authors of future validation studies, who will be faced with many similar issues.

## Quality of comparator instruments

Ideally, it should be clear that comparator instruments validly and reliably measure what they should measure. In practice however, comparator instruments are often not extensively validated, or not validated in the target population. Furthermore, it is unclear when exactly a comparator instrument is “valid”—there are no rules or suggestions about which measurement properties should be of sufficient quality for comparator instruments (and due to a sometimes limited availability of suitable comparator instruments, this may also not be desirable). The most practical approach for reviewers may be to exclude comparator instruments for insufficient quality only if there is no development or validation article available at all.

However, there is one situation in which the quality of an instrument or scale may clearly limit its value as comparator instrument for convergent validity: when the questions of a scale do not all tap into the same construct. Sometimes scales claim to measure a rather “diffuse” topic, such as social functioning. In practice, the questions that comprise one “social functioning” scale often differ greatly from the items of other similarly named scales, and one cannot necessarily assume their scores correlate to a great extent—which is problematic when trying to determine the validity of the instrument under study (for examples of this phenomenon, see Kemmler 1999 [11] or Lacasse 2004 [12]). It may be that “social functioning” is simply not the right construct label for (one of) these scales, or not a precise enough description of the construct, or that the scales have different underlying theories about how to measure social functioning. Another possibility is that they are a collection of questions with different topics around the same general theme rather than one coherent construct. If factor analysis has been performed for the comparator scale, and/or if internal consistency of the scale has been determined, this can help identify scales for which this is the case. We would recommend to look at both the content of the scale and the available information on the measurement properties before deciding to disqualify a comparator scale or instrument due to problems with the coherency of the construct.

In all cases, we would recommend to (briefly) discuss the quality of comparator instruments, as this may help put the results of convergent validity in perspective.

## Suitability of comparator instruments

The construct of the comparator instruments is important for convergent validity: its construct should ideally have a clear relation with the construct under study. This clear relation is not always present for the comparator instruments used in validation studies. Correlation sizes may therefore be hard to predict. An example from our review is the relation between subjective sleepiness and the objective severity of sleep apnea, which is not straightforward [13–15]. If the results from a study disprove any constructed hypothesis, this would do more to illustrate the confusion around the relation between these two constructs, than to provide information about the validity of the instrument. We recommend excluding comparisons with these “unsuitable” constructs from the evidence base.

Furthermore, sometimes comparator constructs are only vaguely related to the construct under study. An example from our review is the relation between sleepiness and quality of life. These constructs are likely somewhat related in patients that suffer from sleep apnea, a condition for which sleepiness is often the main complaint. However, hypotheses of low correlations for weakly related constructs are often correct, and reduce the impact of the hypotheses for more strongly related constructs—which is especially problematic in cases where the former outnumber the latter, and no clear rationale is provided for the choice of these weakly related comparator instruments or domains. We recommend using expected weak correlations only for the requirement that correlations with related constructs are higher than with unrelated constructs.

Sometimes two instruments are employed to validate each other. This is not ideal, as it is unclear which instrument is “at fault” if a hypothesis is not met. However, since it can be quite hard to interpret results either way (see the section “Interpreting outcomes”), reviewers may decide to include these studies and discuss unmet hypotheses in the context of the validation study in question.

## Constructing the hypotheses

COSMIN recommends constructing hypotheses for *relative correlation sizes* of the different comparator instruments. I.e. the correlation of the instrument of interest with instrument A is expected to be higher than its correlation with instrument B. However, the constructs of the comparator instruments may not always be suitable for making meaningful relative hypotheses. To be able to make hypotheses for each comparator instrument, it can be desirable to also construct hypotheses for the absolute magnitude of the correlations. In our review we put each comparator instrument in one of the following categories: either a weak (<0.3), weak to moderate (>0.2 <0.4), moderate (>0.3 <0.7), moderate to high (>0.6 <0.8) or high correlation (>0.7). The overlap between these categories was on purpose, to allow more flexibility in hypotheses. For each correlation we also noted the expected direction of the correlation—positive or negative. Note that we did not focus on the common requirement that convergent validity is adequate if an instrument measuring the same construct is >0.50. We studied the instruments in detail, rather than relying only on the description of the comparator instruments, and when two instruments really measured the same construct we considered a more challenging hypothesis (correlation above >0.70) more adequate.

If an included validation study does use hypotheses to appraise convergent validity, these hypotheses can be integrated with those of reviewers. If the original hypotheses are stricter than as constructed by the reviewers, they can be adjusted. For example, in our review we adjusted a prediction of exactly 0.3 to fit within our “weak to moderate” (>0.2 <0.4) category.

## Interpreting outcomes

When a hypothesis is correct, this contributes to the evidence that the instrument under study measures what it is supposed to measure. However, when a hypothesis is wrong, this can have several causes: (1) the instrument does not measure what it is supposed to measure, (2) the comparator instrument does not measure what it is supposed to measure, or (3) the theory or the assumptions underlying the hypothesis are incorrect [5]. It is not always clear which of these possibilities is true in any given situation, though authors may have their own ideas about the most likely cause. Ideally, possible reasons why a hypothesis was not met are discussed by authors.

Hypotheses about correlations, especially when they measure different but related constructs, are to some extent a best educated guess. A different team of authors or reviewers will likely construct (slightly) different hypotheses, possibly leading to different conclusions. Therefore, we suggest a thorough reporting of the hypotheses that are tested.

## Evidence synthesis

Many systematic reviews about measurement properties report results by synthesis of the evidence. To determine the strength of the evidence for each measurement property, often the number of validation studies studying that measurement property is taken into account, as well as the quality of the studies [8, 16, 17]. The quality of the measurement properties themselves can have evidence that is positive, negative, indeterminate (when only studies of poor quality are available), or conflicting (when results of validation studies are mixed). While this approach makes sense for some measurement properties, for hypothesis testing, the number of studies may be less relevant than the number of hypotheses tested. For example: if there are two studies measuring convergent validity, and one study has only one hypothesis which was found to be inaccurate (negative evidence), and the other has three different hypotheses which are accurate (positive evidence), the scoring method would lead to a “conflicting” overall score. However, 75 % of hypotheses overall are accurate. As such, adding up the hypotheses of the different studies would lead to a more sensible estimation of the convergent validity of an instrument. To incorporate the methodological quality of the different studies in the score one could assign more weight to the hypotheses of better studies, or one could simply decide that the studies all need to be of at least acceptable quality.

## Discussion

Constructing hypotheses for convergent validity in a systematic review requires effort, but is the only way to assess this measurement property if no hypotheses were previously constructed. This article has provided an overview of the issues that can arise in systematic reviews assessing measurement properties of PROMs. Our recommendations are summarized in Box 1.These may be useful for future reviewers and for authors of validation articles with regard to convergent validity as well as other measurement properties that are determined by means of hypothesis testing, such as known-groups validity and discriminant validity (both also subtypes of construct validity) and responsiveness.

The importance of hypothesis testing lies in its ability to help understand the construct the PROM measures. A PROM labeled with an inaccurate construct is a problem which may otherwise remain unrecognized as it does not necessarily affect other measurement properties. Inadequate construct validity leads to the question which construct the PROM does measure, and one will have to look again at the content of the questionnaire, and put this in the context of its comparator instruments. Depending on the situation, either the items of the PROM can be adapted, or it can be decided to re-label the construct the PROM aims to measure.

Establishing convergent validity is prone to several problems: its results depend to an important extent on the choice of comparator instruments and which, and how many, hypotheses are constructed. However, because 75 % of hypotheses need to be accurate rather than all of them, a single inadequate comparator instrument or hypothesis will not immediately prohibit a positive judgment of convergent validity. Furthermore, if results are interpreted critically, we are convinced that an accurate judgment of convergent validity is possible.

## Box1



## Abbreviations

COSMIN: consensus-based standards for the selection of health status measurement instruments
OSA: obstructive sleep apnea
PROM: patient-reported outcome measure
